# Evaluation of the antibacterial effect of hydroalcoholic extract of the galls of *Quercus infectoria* on *Aggregatibacter actinomycetemcomitans*

**DOI:** 10.34172/japid.2023.006

**Published:** 2023-04-05

**Authors:** Zohreh Tabibzadeh Noori, Mohadese Tabatabaei Rad, Mojdeh Hakemi Vala, Mehrdad Karimi, Azadeh Esmaeil Nejad

**Affiliations:** ^1^Department of Periodontics, School of Dentistry, Shahid Beheshti University of Medical Science, Tehran, Iran; ^2^Department of Pediatrics, School of Dentistry, Kerman University of Medical Sciences, Kerman, Iran; ^3^Department of Microbiology, Faculty of Medicine, Shahid Beheshti University of Medical Science, Tehran, Iran; ^4^Faculty of Traditional Medicine, Tehran University of Medical Science, Tehran, Iran

**Keywords:** Aggregatibacter actinomycetemcomitans, Minimum bactericidal concentration, Minimum inhibitory concentration, Quercus infectoria

## Abstract

**Background.:**

*Aggregatibacter actinomycetemcomitans* (Aa) plays a vital role in some destructive forms of periodontitis. While mechanical and chemical plaque control is the first step in periodontitis treatment, side effects of adjunctive chemical agents such as chlorhexidine (CHX) mouthwash have led to the application of natural alternatives with minimal side effects. Therefore, this study evaluated the antibacterial effect of the hydroalcoholic extract of *Quercus infectoria* (Qi) galls on Aa in vitro.

**Methods.:**

The hydroalcoholic extract of Qi was obtained by the maceration method, and Aa bacterial strain was cultured. The inhibition zone diameter was measured through the agar well diffusion method. Also, the minimum inhibitory concentration (MIC) and minimum bactericidal concentration (MBC) values were determined by the broth microdilution method. All the experiments were repeated three times. 0.2% CHX was used as a control.

**Results.:**

The inhibition zone diameter of Aa increased with increasing concentration of Qi extract. While MIC and MBC values for CHX were 0.0039 and 0.0078 mg/mL, respectively, both MIC and MBC values of the Qi extract for this bacterium were similar, i.e., 2.5 mg/mL, which was significantly higherd.

**Conclusion.:**

Since other in vivo studies have confirmed the other properties of this extract and its safety in terms of cytotoxicity and mutagenicity, hydroalcoholic extract of Qi may be used in mouthwashes or local delivery systems to affect periodontal biofilm.

## Introduction

 Periodontal disease is a complex infection caused by the interaction between dental plaque and host immune response, leading to the loss of bone and periodontal tissues if it remains untreated.^1^ There are several bacterial etiologic factors, among which *Agreggatibacter actinomycetemcomitans* (*Aa*), *Porphyromonas gingivalis* (*Pg*), *Prevotella intermedia* (*Pi*), *Bacteroides forsythus* (*Bf*), *Treponema denticola* (*Td*), and *Fusobacterium nucleatum* (*Fn*) are the most important.^2^ *Aa* is a gram-negative non-motile facultative anaerobic *Coccobacillus*, which is the primary etiologic factor in aggressive periodontitis. Also, it has been found in the oral microbiota of patients with chronic periodontitis and periodontally healthy people.^3-5^ *Aa* has seven different serotypes (a to g), among which serotype b is most prevalent in an active periodontal lesion.^6^ This microorganism can remain in periodontal tissues after routine mechanical treatments and cause reinfection of periodontal pockets.^7^ Therefore, chemical agents, including systemic and local antibiotics, were suggested as adjuncts to mechanical therapies in the late 1970s.^7^

 Chlorhexidine (CHX), known as the most effective local antimicrobial mouthwash, is vastly used to prevent and treat periodontal diseases.^8^ Some adverse effects, including discoloration of teeth, composite restorations, burning sensation of the tongue, oral mucosa, temporary taste disturbances, sensitivity, allergic reactions, and in some formulations, nausea and vomiting, have been reported.^2,9^ An increasing rate of antimicrobial resistance and its high cost has led clinicians to use other natural alternatives with minimal side effects, including active peptides, long-chain aldehydes, phenol, ethanol, chloroform, and methanol which have significantly positive therapeutic effects on bacterial, fungal, and viral infections, as well.^10,11^

 Galls of *Quercus infectoria* (*Qi*) are a herbal product obtained from an evergreen bush with the same name, traditionally grown in Greece, western Asia, Iran, and Syria.^12-16^ It has antibacterial, anti-fungal, anti-viral,^17^ anti-inflammatory, and analgesic effects,^18^ helping to control diseases like diabetes, parkinsonism, dysentery, internal hemorrhage, gonorrhea, impetigo, and menorrhagia, with wound-healing effects. It is also an effective local anesthetic and antioxidant agent.^19-23^ The most significant agents in this product are tannins (50%‒70%) with antimicrobial properties,^24,25^ phenolic and flavonoid compounds (2-4 %) like gallotannic acid, gallic acid, and ellagic acid with antibacterial, antioxidant, and anti-viral properties.^24,26-28^

 Several studies have evaluated the effects of *Qi* galls’ extract on oral pathogens and proved its positive effects on *S. mutans*, *S. salivarius*, Pg, Fn,^24^ *S. mutans*, *S. sanguis*,^1^ *E. faecalis*,^29^ and *C. albicans*.^25,26,30,31^ Basri & Fan^32^ and Priya et al^33^ examined aqueous/acetone and methanol extracts of *Qi* galls on some bacteria and found *S. aureus* as the most sensitive genus to it. Basri et al^24^ evaluated the effect of aqueous and acetone extracts of *Qi* galls on some oral bacteria. *S. salivarius* and *Pg* were the most sensitive Gram-positive and negative species, respectively, while the most inhibitory effect was observed with Gram-positive microorganisms. Also, the minimal inhibitory concentration (MIC) and minimal bactericidal concentration (MBC) were reported to be the same for both methanol and acetone extracts.^24^ These findings were confirmed by a study by Lamba et al,^34^ which showed its inhibitory effects on Fn, macrophages, and PMNs. Mohammadi-Sichani et al^35^ compared the ethanol, methanol, and acetone extracts of *Qi *galls on *S. mutans* and reported that the acetone extract had the most significant antibacterial effect.

 However, no study has evaluated the antibacterial effects of *Qi *galls on *Aa* as one of the most critical Gram-negative periodontal pathogens. Also, its impact in terms of MIC and MBC is still unclear. Therefore, the present study aimed to investigate the antibacterial effects of hydroalcoholic extract of *Qi* galls on *Aa* to benefit from this valuable medicinal herb in producing mouthwashes or other antibacterial oral products with herbal origin, improving oral hygiene and preventing infectious periodontal diseases and their recurrence.

## Methods

 This in vitro study was carried out in 2020. Preparation of the plant and Qi galls’ extraction was accomplished in the Department of Traditional Medicine, Tehran University of Medical Sciences, Tehran, Iran, and the antibacterial activity was evaluated in the Microbiology Department, Shahid Beheshti University of Medical Sciences, Tehran, Iran.

###  Preparation of the plant and extraction

 The plant extract was prepared by the maceration technique using distilled water/alcohol (70/30) as the solvent after soaking for 24 hours. The mixture was then filtered using the Whatman filter paper #1. The filtered mixture was thickened in the rotary evaporator device and kept under the biological hood until the rest of the solvent evaporated. Finally, the thoroughly dried extract was stored in a closed sterile container out of light in the refrigerator. The extract’s 20-mg/mL solution was prepared using dimethyl sulfoxide (DMSO) solvent when needed.

###  Microorganism

 The bacterial species used in the present study was a gram-negative facultative anaerobe, *Aggregibacter actinomycetemcomitans* (Aa), Jp2nov99, which was cultured and kept in the microbial collection of the microbiology laboratory in a microaerophilic environment at 5%–10% CO_2_ and 37 °C in the Microbiology Department of Shahid Beheshti University of Medical Sciences.

###  Evaluation of the antibacterial activity 

 The agar-well diffusion and broth microdilution methods were used to evaluate the antibacterial activity of the hydroalcoholic extract of Qi to determine the MIC and MBC values for Aa.

###  Agar well diffusion

 According to the Clinical Laboratory Standard Institute (CLSI) protocol, a bacterial suspension of 0.5 standard McFarland turbidity was prepared using physiologic serum36 so that the estimated number of bacterial cells would be 1.5 × 10^4^ CFU/mL, and the optical density (OD) at 625 nm would be 0.08–0.13. Then, a sterile swab was used to apply a bacterial lawn from the bacterial suspension on the Mueller-Hinton agar culture medium (Merck, Germany). Afterward, 8-mm-diameter wells were produced in the agar gel using a sterile Pasteur pipette. Different concentrations (0.08, 0.15, 0.3, 0.625, 1.25, 2.5, 5, and 10 mg/mL) of the Qi extract were prepared from the 20-mg/mL solution, and 20 µL of each was administered in each agar well. 20 µL of 0.2% chlorhexidine and 20 µL of doxycycline solution (12.5 mg/mL) (Mast, UK) were applied in wells as the positive controls, and 20 µL of DMSO was applied as the negative control.

**Figure 1:**
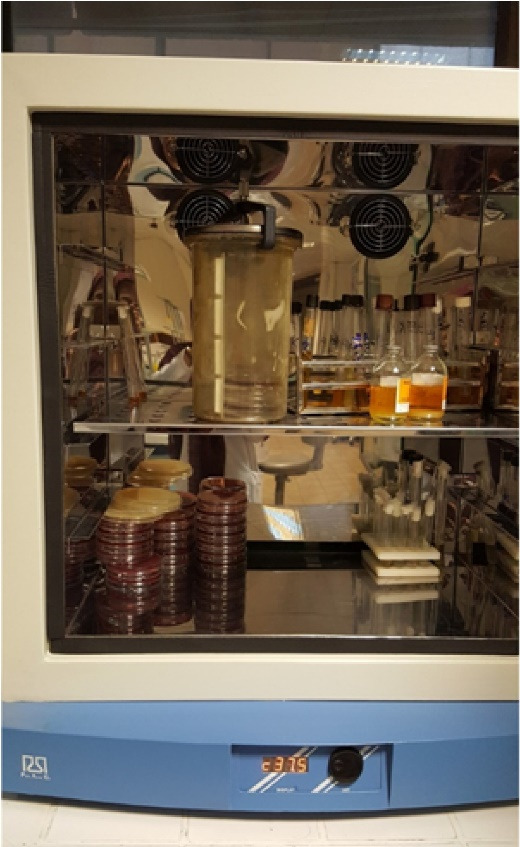
Placing the anaerobic jar in the incubator at 37 °C

**Figure 2:**
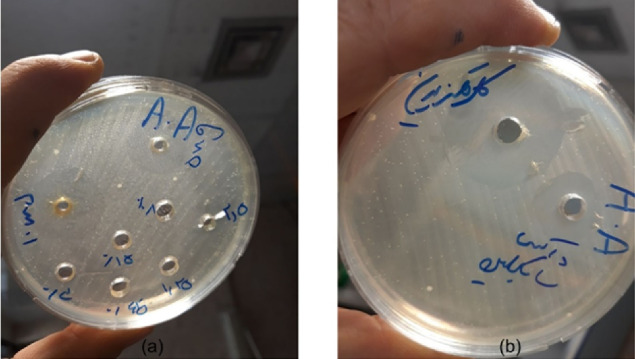
The inhibition zone diameter in the agar well diffusion assay of (a) Qi extract, (b) chlorhexidine and doxycycline

 Agar plates were placed in the anaerobic jar with the GazPak A and kept in a 37 °C incubator. After 24 hours, the plates were retrieved from the jar, and the inhibition zone diameters around each well were measured in mm (Figures 1 and 2). Measurements were repeated in triplicate, and their mean values were reported for case and control groups.

###  Broth microdilution

**Figure 3:**
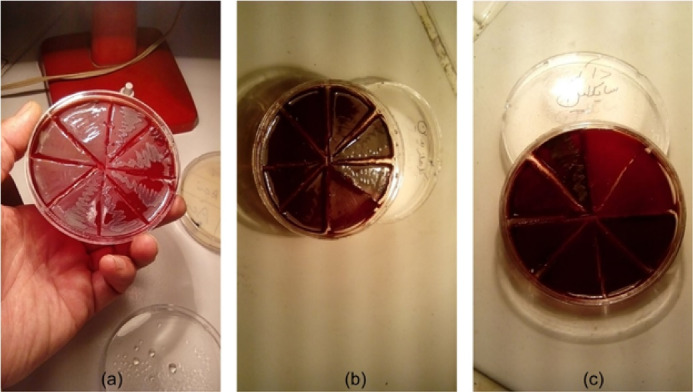
The MIC and MBC of (a) *Qi* extract, (b) chlorhexidine, and (c) doxycycline on *Aa* in broth microdilution assay following the culture on blood agar well plate

 As mentioned above, an 0.5 McFarland standard solution of the Aa bacterial suspension was prepared according to CLSI protocol.^36^ Then, 100 µL of Mueller-Hinton broth was applied to each well in a 96-well plate, followed by adding 100 µL of the 20-mg/mL solution of the Qi extract into the first well. Next, 100 µL of the solution from the first well was mixed in the second well using the sampling technique (pipetage), and this was repeated in the last well so that the concentration of the Qi extract in each well was equal to half of its prior concentration. Finally, 10 µL of 0.5 McFarland standard solution of the Aa bacterial suspension, diluted to 1/20, was added to each well. The same steps were taken with the 100 µL of the 0.2% CHX solution and the 100 µL of the 0.512-mg/mL doxycycline solution in the second and third rows of the plate, respectively, as positive control groups. In the other row, the culture medium, containing only bacterial suspension, and in the last row, the culture medium, containing different concentrations of the extract, were applied to ensure their wholesome. Then, the 96-well plates in the anaerobic jar with the GazPak A were kept in a 37 °C incubator for 24 hours. After incubation, the last clear well was determined for reading the MIC value. To determine the MBC value, an agar culture was made from each of the wells before and after the well was determined as the MIC and incubated in an anaerobic jar overnight. The last clear concentration of the Qi extract with no bacterial growth was determined as the MBC value (Figure 3). The trial was repeated three times, and mean values were reported.

###  Statistical analysis

 Aa’s inhibition zone diameters in the test and the control groups in the agar well diffusion method were measured in millimeters and analyzed with one-way ANOVA using SPSS 21. In addition, the means of the inhibition zone diameters of the microorganism, in the presence of Qi galls and CHX, were compared by the Tukey test. Mean values were considered significant when *P* value was < 0.05.

## Results

 Mean values of the inhibition zone diameters of Aa in plates containing the hydroalcoholic extract of Qi galls were measured at 10.33, 12.83, and 16.83 mm for the concentrations of 2.5, 5, and 10 mg/mL, respectively. There was no inhibition zone in plates containing concentrations of < 2.5 mg/mL of the extract. Also, the mean value for the diameter of the inhibition zone in plates containing 0.2% CHX was 17.13 mm, with 13.13 mm for doxycycline (Table 1). The mean values of MIC and MBC for both the test and control groups are reported in Table 2.

**Table 1:** Comparison of the inhibition zone diameters obtained from the groups (in mm)

		**Experiment repetition**		
		**First**	**Second**	**Third**
Tested materials	*Qi* extract (10 mg/mL)	17	16.5	17
	*Qi* extract (5 mg/mL)	13	12.5	13
	*Qi* extract (2.5 mg/mL)	10	11	10
	*Qi* extract (1.25 mg/mL)	-	-	-
	*Qi* extract (0.625 mg/mL)	-	-	-
	*Qi* extract (0.31 mg/mL)	-	-	-
	*Qi* extract (0.15 mg/mL)	-	-	-
	CHX gluconate (0.2%)	17	17.5	16.9
	Doxycycline (30 µg)	13	12.9	13.5
	DMSO	-	-	-

**Table 2:** Comparison of the MIC and MBC obtained from the groups

**Microorganism**	**MIC (mg/mL)**			**MBC( mg/mL)**		
	***Qi*** **extract**	**CHX**	**Doxycycline**	***Qi *** **extract**	**CHX**	**Doxycycline**
*Aggregatibacter actinomycetemcomitans* (JP2 NOV99)	2.5	0.0039	0.002	2.5	0.0078	0.004

 Pair-wise comparisons of mean values for the inhibition zone diameter by the Tukey test revealed almost comparable values in all the groups with different concentrations of the extract and 0.2% CHX, except for the concentrations of 2.5 and 5 mg/mL of the extract, which was significantly less than 0.2% CHX (*P* < 0.05).

## Discussion

 Recently, the application of various herbal extracts with antimicrobial properties as an oral rinse has attracted the attention of clinicians and periodontal patients, considering the adverse reactions related to chemical mouthwashes.^2,9,37,38^ Some herbal compounds like *Qi* galls have been shown to have high efficiency, with antimicrobial, anti-inflammatory, anti-viral, analgesic, anti-pyrogenic, and antioxidant properties. They are also safe, inexpensive, and traditionally well-accepted.^37-40^ Therefore, the present study evaluated the potential antimicrobial effects of hydroalcoholic extract of *Qi* galls on one of the most critical periodontal pathogens, *Aa*, through agar well diffusion and broth microdilution techniques, which have not been studied before to our knowledge.

 The present study showed the antimicrobial effect of hydroalcoholic extract of *Qi* galls on *Aa*, which can be attributed to some active compounds like phenolics, flavonoids, tannic acid, catechol, rosmarinic acid, pyrogallol, and gallic and ellagic acids, with strong antibacterial, anti-fungal, and antioxidant characteristics.^24,26-28,41^ As several studies have shown, the absolute content of tannins in any form of *Qi* galls extract is high.^24,27-30,34,35,41^ Therefore, this chemical compound is believed to be the main active element responsible for its antimicrobial activity.^25,26,32^ Tannin is a phenolic compound soluble in water and acetone and is precipitated by proteins.^32,42-44^ Recently, a promising aspect in the antibacterial mechanism of *Qi* galls’ extract has been proposed to be the neutralization of the virulence factors, and the quorum sensing in *Pseudomonas* isolated from burn ulcers can inhibit the formation and maturation of the bacterial plaque, especially in the oral cavity.^19^

 Several studies on the effects of *Qi* galls’ extract on some microorganisms have shown that the antibacterial effects of alcoholic and acetone extracts and the antioxidant activity of the aqueous extract are more significant than other solvents.^24-26,28,43-45^ Also, the antibacterial effects of purified compounds of this medicinal herb will increase if mixed with other antioxidant agents.^45^ Nur Syukriah et al^46^ showed that the greatest amounts of bioactive components in *Qi* galls would be extracted if aqueous (80.3%), 70% methanol (74.34%), or 70% ethanol (71.44%) solvents are used. In contrast, the 100% acetone solvent showed the least favorable results, consistent with Mustafa et al,^47^ Tayel et al,^41^ and Wan Nor Amilah et al,^42^ who observed the more efficient extraction of bioactive agents in polarized solvents, leading to higher amounts of gallic and tannic acid in the final aqueous extract.^46^ Therefore, applying both aqueous and alcoholic solvents while preparing the extract of *Qi* galls will lead to a significantly higher antibacterial effect, which was the main reason for choosing this solvent in our study. Therefore, the hydroalcoholic extract of *Qi* galls is widely used, and its polarity has been modified.

 According to other studies, *Qi* galls’ extract has more antibacterial activity against Gram-positive bacteria than Gram-negative ones, probably due to differences in their cell wall structure. In Gram-positive bacteria, the cell wall is made up of peptidoglycans without any outer membrane, making them very vulnerable. In contrast, the outer membrane in Gram-negative bacteria consists of a complex hydrophobic lipopolysaccharide which acts as a strong barrier against any morphological changes in the cell wall.^24,25,32,34,41,42,44^ Comparing our results to those reported by Nagesh et al^29^ confirmed this and will explain the higher MIC and MBC values for *Aa* compared to other Gram-positive bacteria.

 In the present study, the highest mean diameter for the inhibition zone of *Aa *was 16.83 mm for 10% concentration of *Qi* extract, almost similar to that of 0.2% CHX and close to that of *Pg* and *Fn* in the study by Basri et al.^24^ The mean value for MIC and MBC for *Qi* extract in this study was 2.5 mg/mL and significantly higher than that of 0.2% CHX. This difference can be explained by the unique characteristics and diatonic nature of CHX, which enables its rapid, strong, and long-term bonding to surface proteins in Gram-negative bacteria, which maintains its antimicrobial and bacterial agglutinating effects for at least 12 hours in fluid environments like the oral cavity.^48^ On the other hand, differences in the effective concentration for both *Qi* extract and CHX, applied in this study, can explain such a significant discrepancy in their results. It is not unlikely that some changes in the water/ethanol proportion or applying other solvents like methanol or acetone would result in lower values of MIC and MBC for the *Qi* extract. Since no other study is available on the antibacterial effects of *Qi* extract on *Aa*, it is impossible to compare our results to similar investigations; however, its acceptable effects on other common oral bacteria or other periodontal pathogens have been well established.

 Vermani and Prabhat^28^ studied the antibacterial effects of *Qi* extract with different solvents on some oral resident bacteria and reported the highest antibacterial value for the methanol extract on *S. sanguis*. In a similar study, Lamba et al^34^ showed the bacteriostatic effect of methanol and acetone *Qi* extracts on *S. salivarius* and *Pg* and the bactericidal effect on *Fn*. In a study by Basri et al,^24^ MIC and MBC values for methanol and acetone *Qi* extracts against *Fn* was 0.31 mg/mL, MIC for both methanol and acetone *Qi* extracts against *Pg* was 0.63 mg/mL, and MBC for methanol and acetone *Qi* extracts against *Pg* was 1.25 and 2.5 mg/mL, respectively. On the other hand, the mean diameter of the inhibition zone for gram-negatives like *Pg* and *Fn* was 18–19 mm, which is far less than that for gram-positive bacteria and approximately close to that of the present study, which was 17 mm.

 Altogether, discrepancies in the antibacterial effects of *Qi* extract observed in several studies can probably be attributed to different compounds in the fresh herb caused by different geographical growing conditions, the degree of local humidity, the type of solvent, and the different extraction techniques.^25,28,45^ Also, tested concentrations in the broth microdilution assay and the studied microbial species would be a good reason for these controversies. According to Chusri and Voravuthikunchai,^49^ pure fractions of *Qi*, like ethyl acetate and its other principal contents like gallic acid and tannic acid, show a favorable MIC against MRSA, which were 0.06, 0.06, and 0.13 mg/mL, respectively. Therefore, further investigations on the antibacterial effects of the *Qi* extract purified components and comparing it to that of 0.2% CHX as a gold standard seem to be necessary for understanding the antibacterial mechanisms of this valuable herbal medicine.

 Considering the observed antibacterial effects of *Qi* extract on *Aa* in the present study, it is highly suggested to extract the effective components of the whole herb to make further assessments with different solvents in the future. Also, other *in vitro* studies, including the evaluation of cytotoxicity and mutagenicity and its effects on dental plaque biofilm models, are prerequisites for formulating mouthwashes and local delivery agents from *Qi* for clinical use.

## Conclusion

 Hydroalcoholic extracts of Qi may be proper alternatives for chemical antiseptics to treat periodontal disease. Additional studies can further clarify the efficacy of this extract as a mouthwash or a local delivery system to eliminate periodontal biofilms.

## Acknowledgments

 This work was supported by the Dental Research Center, Research Institute of Dental Sciences, School of Dentistry, Shahid Beheshti University of Medical Sciences, Tehran, Iran, with the close contribution of the Department of Traditional Medicine, School of Persian Medicine, Tehran University of Medical Sciences, Tehran, Iran. The authors would like to thank all the technical and scientific support of all technicians and clinicians in both centers. Also, the authors kindly appreciate Dr. Mahshid Namdari for statistical support.

## Availability of Data

 The data used to support the findings of this study are available from the corresponding author upon request.

## Competing Interests

 The authors declare no known competing and financial interests or personal relationships that could have influenced the work reported in this paper.

## Ethical Approval

 This cross-sectional study was approved by the Ethics Committee of the Dental School, Shahid Beheshti University of Medical Sciences, Tehran, Iran (IR.SBMU.RIDS.1396.498).

## Funding

 This study was performed in Shahid Beheshti University of Medical Sciences.
